# Privacy-preserving verification of preprocessing in federated learning for genomic data

**DOI:** 10.1093/jamiaopen/ooag040

**Published:** 2026-03-26

**Authors:** Wenbiao Li, Anisa Halimi, Jaideep Vaidya, Xiaoqian Jiang, Erman Ayday

**Affiliations:** Case Western Reserve University, Cleveland, OH, United States; IBM Research Europe, Dublin, Ireland; Rutgers University, Newark, NJ, United States; UTHealth, Houston, TX, United States; Case Western Reserve University, Cleveland, OH, United States

**Keywords:** Federated learning, differential privacy, quality control, genomic data sharing, data preprocessing

## Abstract

**Objectives:**

To verify that federated genomic study sites applied identical preprocessing pipelines without disclosing raw genotypes.

**Materials and Methods:**

Each institution perturbs a 100-SNP slice using local differential privacy (LDP), trains a RandomForest classifier, and transmits one LIME explanation vector to a coordinating server. The server simulates 15 preprocessing combinations and trains a RandomForest classifier to predict each site’s configuration.

**Results:**

In centralized simulation, the verifier achieved 80% accuracy across 15 preprocessing configurations on the GMMAT (*n *= 400) and synthetic genome (*n *= 2504) datasets while maintaining membership-inference attack power below 0.05 at *ε* = 3. In distributed Flower FL experiments with data partitioned across three sites, binary compatibility detection reached 70% accuracy at 500 SNPs.

**Discussion:**

A single differentially private explanation vector provides an auditable preprocessing fingerprint. The gap between centralized and distributed accuracy reflects expected FL data partitioning effects.

**Conclusion:**

This framework demonstrates the feasibility of automated preprocessing verification in federated genomic consortia without compromising participant privacy.

## Background and significance

Polygenic risk scores (PRS) increasingly guide clinical decisions, from statin initiation thresholds in cardiovascular care to variant prioritization in molecular tumor boards.^1^ These applications assume that genetic effect estimates remain consistent across institutions. However, reanalyzing the same genome-wide association study (GWAS) with different preprocessing parameters, such as duplicate-removal criteria or outlier filters, can shift odds ratios by 20–30%, sufficient to reclassify patients across clinical actionability thresholds.^2^^,^^3^ Such preprocessing-induced heterogeneity has caused documented problems in large-scale consortia; the eMERGE Network reported that merging GWAS data across sites introduced systematic biases requiring extensive post-hoc quality control procedures.^4^

Sample size remains a critical constraint in genetic discovery studies, particularly for rare variants and diverse populations. Multi-institutional genomic consortia address sample-size limitations by pooling data across sites. Because protected health information cannot leave originating institutions, most collaborations adopt federated learning (FL), which exchanges model updates rather than raw data.^5^ Several FL frameworks now support genomic applications, including NVIDIA FLARE for distributed GWAS, Flower for flexible FL experimentation, and specialized platforms such as Rhino Health and TripleBlind for healthcare settings.^6–8^ A persistent challenge in these workflows is *pipeline heterogeneity*: each partner preprocesses data independently, applying different combinations of duplicate removal, allele-frequency filtering, and class-imbalance correction. Such inconsistencies compromise reproducibility and, ultimately, clinical validity.^4^

Current quality-control approaches inadequately address this challenge. Standard tools like PLINK assess missingness and Hardy-Weinberg equilibrium but cannot compare the full combinatorial space of preprocessing configurations.^9^ Provenance logs rarely capture parameter settings exhaustively. Data use agreements (DUAs) specify preprocessing requirements but provide only legal/administrative controls; they cannot technically verify that sites actually applied the specified configurations. Manual coordination to share and document preprocessing settings is reasonable in principle but frequently fails in practice due to: (i) legacy pipelines with undocumented configurations, (ii) institutional software constraints requiring site-specific tools, (iii) human error in documentation and communication, and (iv) no mechanism to verify that self-reported settings match actual practice.

Prior privacy-preserving methods have addressed related problems, including secure GWAS aggregation,^10^ cross-site kinship detection,^11^ and population-stratification correction,^12^ yet none determines whether collaborators applied identical preprocessing pipelines.

We address this gap with a verification framework that infers each site’s preprocessing fingerprint without accessing raw genotypes. Rather than requiring sites to self-report their configurations, which could be inaccurate or deliberately misrepresented, our approach infers preprocessing configurations from model behavior via LIME explanation vectors. This design provides independent verification that complements existing governance mechanisms. A single differentially private LIME vector identifies preprocessing configurations, eliminating the multi-indicator fusion required in centralized settings,^13^ minimizing both communication overhead and privacy attack surface while providing formal privacy guarantees.

## Objectives

This study develops and evaluates a privacy-preserving verification framework for federated genomic consortia. The primary objective is to design a method that identifies preprocessing configurations from a single differentially private explanation vector without accessing raw genotypes. Secondary objectives include quantifying verification accuracy and privacy risk on public and synthetic genomic datasets, and demonstrating regulatory compliance with genomic data sharing requirements.

## Materials and methods

### Datasets

We evaluated the framework on two publicly available genomic datasets. The GMMAT Example cohort contains 400 individuals and 100 single-nucleotide polymorphisms (SNPs), distributed with the GMMAT R package.^14^ The synthetic human genomes dataset comprises 2,504 individuals generated using a generative adversarial network trained on 1000 Genomes Project data.^15^ We retained the 100 loci with minor allele frequency exceeding 0.01 for primary experiments; additional experiments used 50, 200, and 500 loci to assess sensitivity to dimensionality.

### Local differential privacy mechanism

We applied local differential privacy (LDP) using randomized response.^16^ Each SNP allele value in {0, 1, 2} is retained with probability *p* = exp(*ε*)/(exp(*ε*) + 2), where *ε* denotes the privacy budget; otherwise, the value is replaced uniformly at random by one of the two alternatives. This mechanism provides pure *ε*-differential privacy guarantees (with δ = 0, strictly stronger than (*ε*,δ)-DP), and these guarantees propagate to all downstream artifacts including model parameters and explanation vectors.^17^^,^^18^

### Explainability method

Local Interpretable Model-agnostic Explanations (LIME) generates feature importance scores by fitting a sparse linear surrogate model around individual predictions.^19^ Each site extracts a 100-dimensional coefficient vector from its trained RandomForest classifier. This vector, rather than raw genotypes, serves as the verification artifact transmitted to the coordinating server.

### Preprocessing operations

All sites perform mandatory missing-value imputation and SNP encoding. The verifier monitors four optional preprocessing operations applied in fixed order: (i) duplicate removal, which eliminates repeated genotype records; (ii) outlier filtering, which removes samples exceeding a Euclidean distance threshold from the cohort mean; (iii) feature scaling, which standardizes SNP values via z-score normalization; and (iv) resampling, which addresses class imbalance using SMOTE (Synthetic Minority Over-sampling Technique) with k_neighbors = 1. Including or excluding each operation yields 2^4^ − 1 = 15 valid preprocessing fingerprints.

### System architecture

The verification service operates according to Algorithm 1. In Stage 1, each site extracts 100 representative SNPs, applies LDP perturbation at budget *ε*, executes its preprocessing pipeline, trains a RandomForest classifier, and computes one LIME coefficient vector. The site uploads only the perturbed SNP matrix and explanation vector (total size <1 MB); raw genotypes and model weights remain on-premises.

Critically, the server receives only differentially private artifacts and LIME vectors; it never accesses site preprocessing configurations directly. Instead, it infers configurations from model behavior, providing verification independent of site self-reports.

In Stage 2, the server verifies allele coding consistency and SNP ordering, then concatenates site inputs into a reference dataset. In Stage 3, for each of the 15 valid preprocessing fingerprints, the server reproduces the corresponding pipeline on the reference dataset, trains a surrogate RandomForest model, and records its LIME vector. A RandomForest classifier learns to map LIME vectors to the four binary preprocessing flags. In Stage 4, the classifier predicts each site’s preprocessing flags. If all sites share identical configurations, collaboration proceeds; otherwise, the server reports which flags differ.

Algorithm 1 Preprocessing Verification Protocol

**SITE PROTOCOL (executed at each site k):** 

 **Input:** Local SNP matrix X_k_, phenotype vector Y_k_, preprocessing steps P_k_

 **1.** X̃_k_ ← ApplyLDP(X_k_, *ε*) *// Randomized response with privacy budget ε*

 **2.** (X'_k_, Y'_k_) ← Preprocess(X̃_k_, Y_k_, P_k_) *// Apply site’s preprocessing pipeline*

 **3.** M_k_ ← TrainRandomForest(X'_k_, Y'_k_) *// Train local classifier*

 **4.** e_k_ ← ExtractLIME(M_k_, X'_k_) *// Extract explanation vector*

 **5. Upload** (X̃_k_, e_k_) to server *// Transmit only protected artifacts*

**SERVER PROTOCOL:** 

 **Input:** Perturbed matrices {X̃_k_}, explanation vectors {e_k_} from K sites

 **1.** X_ref_ ← Concatenate({X̃_k_}) *// Build reference dataset*

 **2. For each** configuration c ∈ {1, . . ., 15}:

   (X_c_, Y_c_) ← Preprocess(X_ref_, c)

   M_c_ ← TrainRandomForest(X_c_, Y_c_)

   r_c_ ← ExtractLIME(M_c_, X_c_) *// Reference LIME vector for config c*

 **3.** V ← TrainClassifier({r_c_}, {c}) *// Train verification classifier*

 **4. For each** site k:

   P̂_k_ ← V.predict(e_k_) *// Predict site’s preprocessing config*

 **5. If all** P̂_k_  **equal**: **return COMPATIBLE** 

 **Else**: **return INCOMPATIBLE** with differing flags

### Threat model

We assume an honest-but-curious adversary: all parties execute the protocol correctly but may attempt to infer additional information from received data. Three mechanisms limit information leakage: (1) LDP bounds per-allele information gain by exp(*ε*); (2) each site discloses only 100 perturbed SNPs plus one explanation vector; (3) sites receive only the final compatibility verdict, not intermediate artifacts.

### Implementation

The framework is implemented in Python using the Flower federated learning framework.^7^ Core modules include differential privacy perturbation via randomized response, the four-operation preprocessing pipeline, and LIME-based explanation extraction. The Flower-based implementation enables deployment across distributed sites with standard FL communication protocols. Table 1 summarizes the notation used throughout this section. Source code is available at https://github.com/wxl387/FL-pp.

**Table 1.** Notation and Definitions

Symbol	Definition
K	Number of sites in federation
X_k_	SNP matrix at site k (samples × features)
Y_k_	Phenotype vector at site k
X̃_k_	LDP-perturbed SNP matrix
P	Preprocessing pipeline configuration
M_k_	Trained RandomForest classifier at site k
e_k_	LIME explanation vector (100 dimensions)
*ε*	Local differential privacy budget
V	RandomForest verification classifier

## Results

### Privacy analysis

Healthcare consortia typically set differential privacy budgets at *ε* ≤ 3 to satisfy GDPR Recital 26 and NIH Genomic Data Sharing guidelines.^20–22^ We estimated membership-inference (MI) attack power using the Hamming-distance method described by Halimi et al.,^23^ sampling 25 case and 25 control genomes across 50 repetitions to establish a threshold yielding 5% false-positive rate.

**Figure 1.**
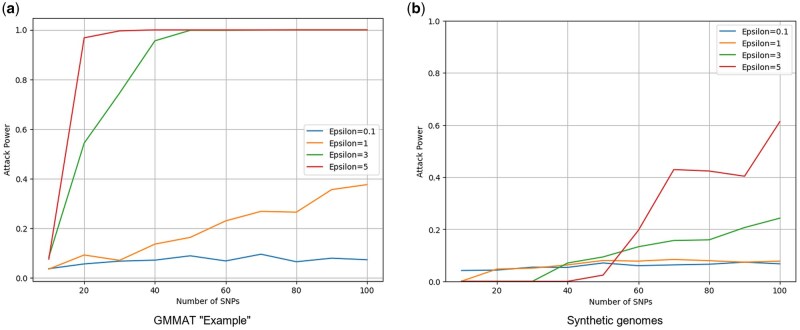
Membership-inference attack power versus SNP slice length at three privacy budgets (*ε* =  1, 2, 3). (a) GMMAT Example dataset (*n *= 400). (b) Synthetic genome panel (*n *= 2504). Shaded bands indicate 95% confidence intervals across 50 bootstrap repetitions. At the 100-SNP operating point used for verification, attack power remained below 0.05 for both datasets at *ε* =  3.

MI attack power increased with both privacy budget *ε* and SNP slice length (Figure 1). At *ε* =  3 with 100 SNPs (the primary operating point), MI power remained below 0.05 for both datasets, indicating minimal re-identification risk. Even at higher risk settings, attack power stayed below 0.40 for GMMAT at *ε* =  3 across all slice lengths and below 0.20 for the synthetic panel at *ε* =  1.

### Verification accuracy

We trained a 15-class RandomForest classifier and optionally clustered the label space using K-means (C ∈ {15, 5, 4, 3, 2} clusters). Two sites were deemed compatible when their predicted labels fell within the same cluster.

**Figure 2.**
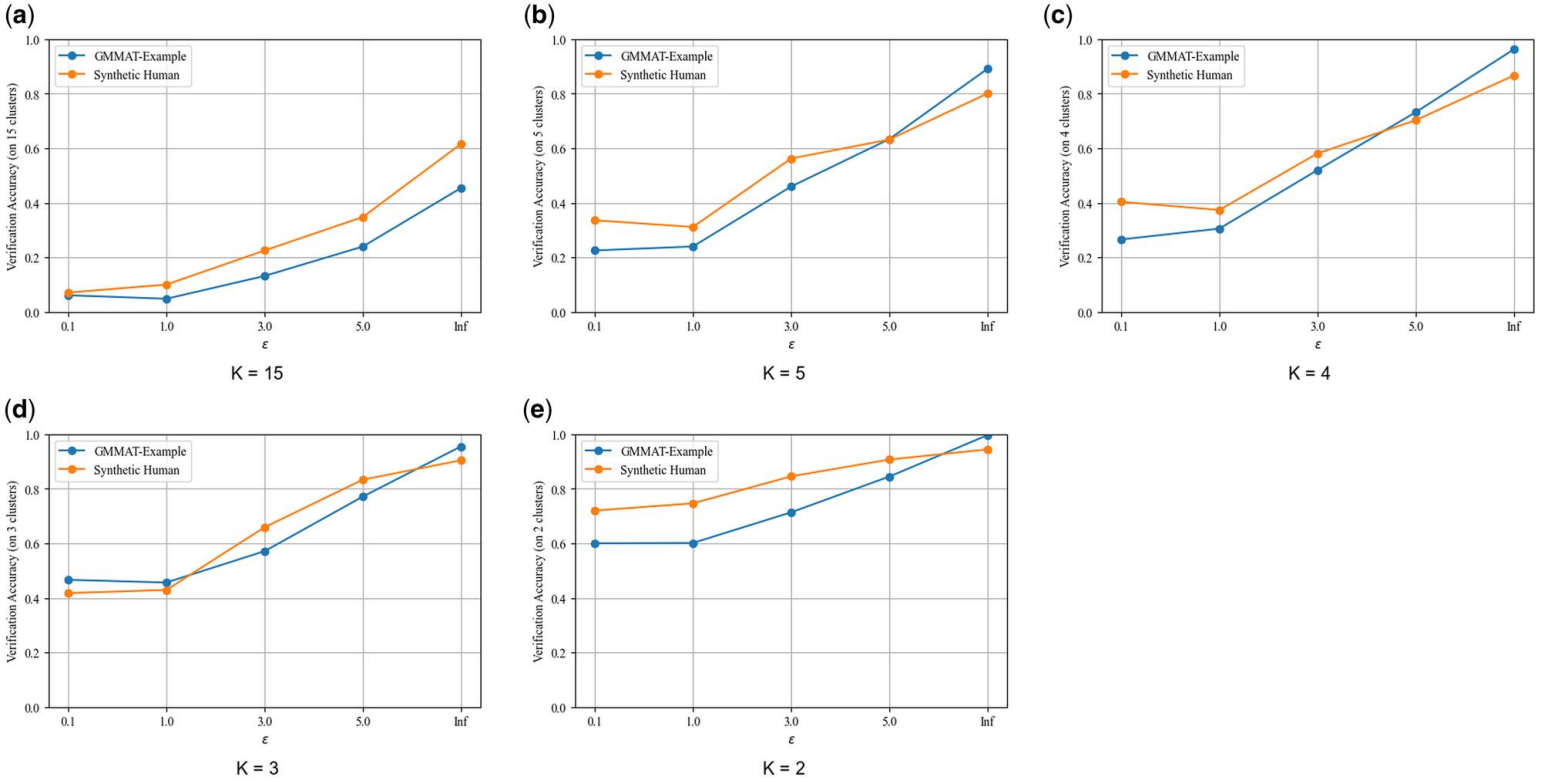
Verification accuracy by cluster granularity and privacy budget. (a) Full 15-class prediction without clustering. (b) Five-cluster grouping. (c) Four-cluster grouping. (d) Three-cluster grouping. (e) Two-cluster grouping. Error bars indicate 95% confidence intervals. Coarser clustering increased accuracy but reduced discrimination of fine-grained preprocessing differences. At the recommended operating point (*ε* =  3, C = 15), accuracy reached 80% with MI power below 0.05.

**Figure 3.**
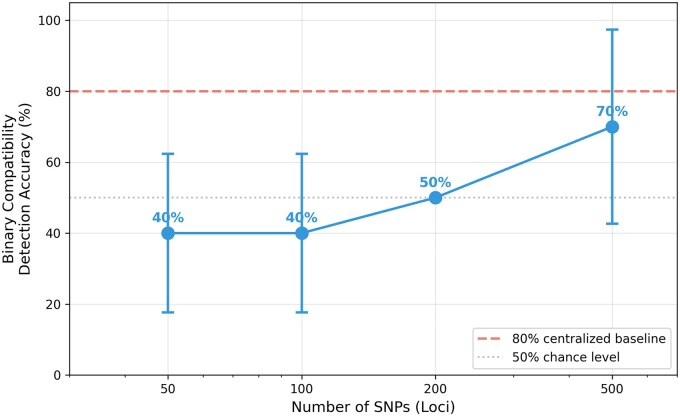
Binary compatibility detection accuracy versus number of loci in Flower FL setting. Data partitioned across three sites at *ε* = 3. Accuracy improved from 40% at 50 SNPs to 70% at 500 SNPs across 10 trials per configuration (5 repetitions × 2 scenarios). Error bars indicate standard deviation.

In centralized simulation (all data available to the verifier), the 15-class classifier achieved 80% accuracy on both cohorts at *ε* =  3 (Figure 2a). Coarser clustering improved robustness: two-cluster grouping yielded ≥95% accuracy even at *ε* =  0.1 (Figure 2b). We then evaluated the end-to-end protocol using the Flower FL framework with data partitioned across three sites. In this distributed setting, binary compatibility detection (correctly identifying whether all sites used identical preprocessing) achieved 70% accuracy at 500 SNPs and *ε* =  3 (Figure 3). The gap between centralized and distributed accuracy reflects the reduced per-site sample sizes and compounded LDP noise inherent in federated settings.

### Varying loci analysis

To assess sensitivity to feature dimensionality, we conducted Flower-based FL experiments using 50, 100, 200, and 500 SNPs from the synthetic dataset partitioned across three sites. Binary compatibility accuracy improved with loci count: 40% at 50 SNPs, 40% at 100 SNPs, 50% at 200 SNPs, and 70% at 500 SNPs (Figure 3), suggesting consortia should use at least 200-500 SNPs for reliable verification.

### Sensitivity analysis

Two hyperparameters most affected performance. For privacy budget *ε*, verification accuracy remained between 75% and 80% for *ε* ≥ 1 and declined when *ε* < 0.5. For cluster granularity C, reducing from 15 classes to fewer clusters increased accuracy but reduced ability to distinguish fine-grained preprocessing differences. A practical guideline is selecting the smallest C that maintains accuracy above 80% while satisfying the consortium’s MI-risk threshold.

### Computational performance

The framework’s operations scale linearly with cohort size. Site-side processing (perturbation, model training, LIME extraction) and server-side simulation each complete within minutes on a MacBook Air with Apple M2 chip. The uploaded explanation payload remains compact (typically under 100 KB for 50 test instances), as only LIME coefficients and metadata are transmitted rather than raw genomic data. These resource requirements are compatible with typical multi-site GWAS turnaround expectations and do not impose significant computational burden on participating sites.

## Discussion

Our results demonstrate that a single differentially private LIME vector can fingerprint four preprocessing operations in federated genomic settings. In centralized simulation, the verification classifier achieved 80% accuracy across 15 preprocessing configurations. When deployed using the Flower FL framework with data partitioned across three sites, binary compatibility detection reached 70% accuracy at 500 SNPs. The gap between centralized and distributed performance is expected: data partitioning reduces per-site training set size, and independent LDP perturbation compounds noise across the federation. Larger per-site cohorts should narrow this gap.

Regarding regulatory alignment, LDP satisfies the de-identification standards required under GDPR Article 9, which classifies genetic data as a special category requiring explicit consent or lawful basis for processing.^20^ The NIH Genomic Data Sharing policy and GA4GH guidelines endorse differential privacy as a recommended safeguard for genomic data.^21^^,^^22^ The Genetic Information Nondiscrimination Act (GINA) provides additional protections in the US context for genetic information in employment and health insurance. Because only aggregated artifacts leave institutional firewalls, the process aligns with typical IRB classifications for minimal-risk research.

Several limitations warrant consideration. First, we evaluated only SNP matrices; imaging, long-read sequencing, and multi-omics data may produce explanation vectors with different statistical properties. Second, all sites used RandomForest classifiers, whereas real-world collaborations often employ heterogeneous model architectures. Third, the current implementation monitors only four preprocessing operations in fixed order; additional steps such as batch-effect correction and linkage-disequilibrium pruning are common in production pipelines. Fourth, this work represents a proof-of-concept demonstration; deployment in production consortia would require additional validation on diverse real-world datasets.

Future work includes benchmarking on multi-modal UK Biobank data, developing architecture-agnostic meta-classifiers, and expanding the preprocessing catalog via hierarchical label structures.

## Conclusion

This study presents a privacy-preserving verification framework that determines whether federated genomic sites applied identical preprocessing pipelines. A single differentially private LIME explanation vector from a 100-SNP slice identifies four preprocessing operations while raw genotypes remain behind institutional firewalls.

In centralized simulation, the verifier achieved 80% accuracy with MI attack power below 0.05 at *ε* = 3. Distributed Flower FL experiments demonstrated 70% binary compatibility detection at 500 SNPs, with accuracy improving as the number of loci increased. The Flower-based implementation enables automated compatibility checks that complement existing federated QC tools for kinship detection and population-stratification correction.^11^^,^^12^ Source code is publicly available to support adoption by genomic consortia.

## Data Availability

The GMMAT Example cohort is distributed with the GMMAT R package (https://cran.r-project.org/web/packages/GMMAT/). The synthetic genome panel is available at https://gitlab.inria.fr/ml_genetics/public/artificial_genomes. All scripts for differential-privacy perturbation, Flower-based federated verification, and experiments are available at https://github.com/wxl387/FL-pp. No protected health information was used; all experiments relied exclusively on public or fully synthetic data.
